# Multiple Origins and Nested Cycles of Hybridization Result in High Tetraploid Diversity in the Monocot *Prospero*

**DOI:** 10.3389/fpls.2018.00433

**Published:** 2018-04-06

**Authors:** Tae-Soo Jang, John S. Parker, Khatere Emadzade, Eva M. Temsch, Andrew R. Leitch, Hanna Weiss-Schneeweiss

**Affiliations:** ^1^Department of Botany and Biodiversity Research, University of Vienna, Vienna, Austria; ^2^Cambridge University Botanic Garden, Cambridge, United Kingdom; ^3^Queen Mary College, University of London, London, United Kingdom

**Keywords:** allopolyploidy, autopolyploidy, genome evolution, nested cycles of hybridization, numerical chromosomal variation, *Prospero autumnale* complex

## Abstract

Polyploidy is a major driving force in angiosperm evolution, but our understanding of establishment and early diversification processes following allo- vs. auto-polyploidy is limited. An excellent system to address such questions is the monocot plant *Prospero autumnale*, as it comprises several genomically and chromosomally distinct diploid cytotypes and their auto- and allotetraploid derivatives. To infer origins and evolutionary trajectories of the tetraploids, we use genome size data, *in situ* hybridization with parental genomic DNAs and specific probes (satDNA, rDNAs), as well as molecular-phylogenetic analyses. Thus, we demonstrate that an astounding range of allotetraploid lineages has been formed recurrently by chromosomal re-patterning, interactions of chromosomally variable parental genomes and nested cycles of extensive hybridization, whereas autotetraploids have originated at least twice and are cytologically stable. During the recurrent formation and establishment across wide geographic areas hybridization in some populations could have inhibited lineage diversification and nascent speciation of such a hybrid swarm. However, cytotypes that became fixed in populations enhanced the potential for species diversification, possibly exploiting the extended allelic base, and fixed heterozygosity that polyploidy confers. The time required for polyploid cytotype fixation may in part reflect the lag phase reported for polyploids between their formation and species diversification.

## Introduction

Polyploidy is found in the ancestry of all lineages (Van de Peer et al., 2009), and is particularly important in the diversification and speciation of flowering plants (Wendel, 2000; Soltis et al., 2009; Weiss-Schneeweiss et al., 2013). Our understanding of the early establishment and diversification processes of allopolyploids has been driven by ground breaking work from a number of allopolyploid systems, e.g., in *Tragopogon* (Chester et al., 2012), *Spartina* (Ainouche et al., 2012), *Nicotiana* (Renny-Byfield et al., 2013), *Cardamine* (Mandáková et al., 2013), or *Mimulus* (Vallejo-Marin et al., 2015). Less is known about autopolyploid establishment and diversification, despite autopolyploidy being at least as common as allopolyploidy (Barker et al., 2016). Assessing potential differences with respect to establishment and early diversification between autopolyploids and allopolyploids requires a group of closely related, yet distinct diploid, autopolyploid and allopolyploid lineages that may or may not be recognized taxonomically.

An excellent system to elucidate genome dynamics and polyploid evolutionary trajectories is provided by the monocot *Prospero autumnale* (Hyacinthaceae, autumn squill), distributed across the Mediterranean Basin, Europe, and western Asia (Parker et al., 1991; Speta, 1993). The *P. autumnale* complex is remarkably variable in chromosome number (dysploidy on diploid level, polyploidy, B-chromosomes: Ainsworth et al., 1983; Vaughan et al., 1997; Jang et al., 2013, 2016), chromosome structure (fusions, inversions, translocations, centric shifts, supernumerary chromosomal segments: Taylor, 1997; Jang et al., 2013), genome size (Ebert et al., 1996; Vaughan et al., 1997; Jang et al., 2013) and repetitive DNA distribution and copy number (Emadzade et al., 2014). The *P. autumnale* complex encompasses four diploid cytotypes with unique combinations of basic chromosome number (*x* = 7, 6, 5), genome size, locations of pericentric satellite DNA *PaB6* and of 5S and 35S rDNA loci (Jang et al., 2013; Emadzade et al., 2014). Sharing the same chromosome number, *x* = 7, cytotype AA differs from cytotype B^7^B^7^ by its larger genome. Both, cytotype B^6^B^6^, with *x* = 6 and an intermediate genome size, cytotype B^5^B^5^, with *x* = 5 and the smallest genome size, have originated independently from an ancestral karyotype (*x* = 7) via chromosomal translocations (referred to as “fusions” due to the lack of explicit evidence of the type of translocation; Jang et al., 2013). For more information please see below (Study Group).

Polyploidy is rampant in the *P. autumnale* complex, and both autopolyploids (i.e., polyploids involving only one of the genomically and chromosomally distinct diploid cytotypes) and allopolyploids (i.e., polyploids involving two of these diploid cytotypes) have been described (Ainsworth et al., 1983; Speta, 1993; Vaughan et al., 1997). The polyploids are sympatric with their diploid progenitors, at least over part of their ranges, but most have undergone expansion beyond their parental limits (Parker et al., 1991; Vaughan et al., 1997).

Here we examine the origins and evolutionary trajectories of closely related autotetraploids and allopolyploids, involving three diploid cytotypes of *Prospero*. To this end, we use genome size measurements as well as *in situ* hybridization with probes of parental genomic DNA, satellite DNA *PaB6*, and 5S and 35S rDNAs and interpret those within an established phylogeny. Specifically, we aim to (1) cytologically characterize tetraploid lineages within the *P. autumnale* complex; (2) elucidate genomic evolution in tetraploids with respect to genome size and repetitive DNAs in comparison to their parental diploids; and (3) disentangle the evolutionary histories of tetraploids. By elucidating genome evolution and early polyploid diversification in a group of closely related lineages we want to contribute to a better understanding of the astonishing success of polyploidy in angiosperm divergence.

## Materials and methods

### Study group

The *P. autumnale* complex is distributed across the Mediterranean Basin, Europe and western Asia (Parker et al., 1991; Speta, 1993). It encompasses four diploid cytotypes: AA, B^7^B^7^, B^6^B^6^, and B^5^B^5^ (Jang et al., 2013; Table 1). Cytotype AA occurs in western Mediterranean and has basic chromosome number of *x* = 7 (2*n* = 14) and largest genome size. Cytotype B^7^B^7^ is widespread in whole Mediterranean region, possesses 2*n* = 14 chromosomes and medium sized genome (Table 1). Karyotype structure of AA and B^7^B^7^ cytotypes is similar, with one locus of 35S rDNA in chromosome 3, but they differ in size of chromosomes and the location of 5S rDNA locus (chromosome 2 and chromosome 1, respectively). Additionally, two well-defined chromosomal lineages can be distinguished within B^7^B^7^ cytotype, one with single and one with duplicated locus of 5S rDNA in long arm of chromosome 1. Cytotypes B^6^B^6^ and B^5^B^5^ (*x* = 6 and 5, respectively) originated independently from within cytotype B^7^B^7^. Cytotype B^6^B^6^ occurs on Crete, has genome size larger than that of the B^7^B^7^ cytotype, one locus of 35S rDNA in chromosome 3 and two loci of 5S rDNA (in chromosomes 1 [5S^1^ rDNA] and 2 [5S^2^ rDNA]). The base chromosome number of *x* = 6 originated via translocations involving chromosomes 6 and 7 of cytotype B^7^B^7^ giving rise to large submetacentric “fusion” chromosome (F^1^(6-7); Jang et al., 2013). Cytotype B^5^B^5^ occurs in Libya, but is not involved in polyploid formation, and thus will not be discussed here in detail (Jang et al., 2013). All diploid cytotypes differ also in the copy number and loci number of pericentric *PaB6* satellite DNA (Emadzade et al., 2014; Table 1).

**Table 1:** Number of 5S rDNA, 35S rDNA and satellite DNA *PaB6* signals, genome size, ITS ribotypes and cp haplotypes in diploids and tetraploids of the *P. autumnale* complex.

**Cytotype**	**2*n***	**Number and origin of 35S and 5S rDNA loci per diploid genome^‡^**			**cp haplotype/ITS ribotype^§^**	**Number and strength of *PaB6* signals (per 2*n*)**	**Genome size 1C (pg) ± SD**
		**35S^3^**	**5S^1^**	**5S^2^**			
**DIPLOIDS^†^**							
AA	14	2 A	–	2 A	A/A	2 weak	7.85 ± 0.05
B^7^B^7^ (type I)	14	2 B^7^	2 Type I	–	B^7^/B^7^	12–14 moderate	4.23 ± 0.05
B^7^B^7^ (type II)	14	2 B^7^	2 Type II	–	B^7^/B^7^	6 weak	4.45 ± 0.02
B^6^B^6^	12	2 B^6^	2 B^6^	2 B^6^	B^6^/B^6^	12 strong	6.27 ± 0.08
**POLYPLOIDS**							
**B**^7^**B**^7^**B**^7^**B**^7^							
H132	28	4 B^7^	4 Type I	–	B^7^/B^7^	15–26 weak	8.22 ± 0.04
H172	28	4 B^7^	4 Type I	–	B^7^/B^7^	15–26 weak	9.14 ± 0.05
H401	28	4 B^7^	4 Type I	–	B^7^/B^7^	15–26 weak	9.50 ± 0.18
H435	28	4 B^7^	4 Type I	–	B^7^/B^7^	15–26 weak	9.07 ± 0.03
H534	28	4 B^7^	4 Type I	–	B^7^/B^7^	15–26 weak	8.53 ± 0.05
H577	28	4 B^7^	4 Type I	–	B^7^/B^7^	15–26 weak	9.00 ± 0.11
H615	28	4 B^7^	4 Type I	–	B^7^/B^7^	15–26 weak	9.29 ± 0.01
H628	28	4 B^7^	4 Type I	–	B^7^/B^7^	15–26 weak	–
H230	28	4 B^7^	4 Type II	–	B^7^/B^7^	1 strong distal + few weak	–
H310	28	4 B^7^	4 Type II	–	B^7^/B^7^	2 strong distal + few weak	7.45 ± 0.03
**AAB**^7^**B**^7^							
H603^¶^	28	2 B^7^	2 Type I	2 A	B^7^/B^7^	12–14 weak	12.70 ± 0.09
H607^¶^	28	2 B^7^	2 Type I	2 A	B^7^/B^7^	12–14 weak	13.10 ± 0.16
**B**^6^**B**^6^**B**^7^**B**^7^							
GROUP I							
H153^¶^	25	4 B^allo^	4 B^allo^	4 B^allo^	B^6^/B^6^	23 strong + 2 moderate	12.17
H208	25	4 B^allo^	4 B^allo^	4 B^allo^	B^6^/B^6^	22 strong + 3 moderate	12.10 ± 0.05
H14^¶^	26	4 B^allo^	4 B^allo^	4 B^allo^	B^6^/B^6^	24 strong + 2 moderate	11.81 ± 0.37
H96^¶^	26	4 B^allo^	4 B^allo^	4 B^allo^	B^6^/B^6^	22 strong + 4 moderate	11.67 ± 0.01
H207	27	4 B^allo^	4 B^allo^	4 B^allo^	B^6^/B^6^	25 strong + 2 weak	12.00 ± 0.01
H300^¶^	28	4 B^allo^	4 B^allo^	4 B^allo^	B^6^/B^6^	26 strong + 2 weak	11.61 ± 0.02
H331	28	4 B^allo^	4 B^allo^	4 B^allo^	B^6^/B^6^ & B^7^	28 strong	11.53
GROUP II							
H356	28	2 B^7^	2 Type II + 2 B^allo^	2 B^allo^	B^6^/B^7^	14 strong	9.82 ± 0.25
H363^¶^	28	2 B^7^ + 1 weak B^allo^	2 Type II + 2 B^allo^	2 B^allo^	B^6^/B^7^	14 strong	10.25 ± 0.05
H388	28	2 B^7^ + 1 weak B^allo^	2 Type II + 2 B^allo^	2 B^allo^	B^6^/B^7^	14 strong	10.25 ± 0.03
H410^¶^	28	2 B^7^ + 1 weak B^allo^	2 Type II + 2 B^allo^	2 B^allo^	B^7^/B^7^	14 strong	10.32 ± 0.06
H434^¶^	28	2 B^7^	2 Type II + 2 B^allo^	2 B^allo^	B^7^/B^7^	14 strong	10.06 ± 0.20
GROUP III							
H238^¶^	28	1 B^7^ + 2 B^allo^ + 1 weak B^allo^	1 Type II + 3 B^allo^	3 B^allo^	B^6^/B^6^ & B^7^	21 strong	10.84 ± 0.07
GROUP IV							
H152^¶^	28	3 B^7^ + 1 weak B^allo^	2 Type I + 1 B^allo^ + 1 Type II	1 B^allo^	B^7^/B^7^	7 strong	9.25 ± 0.01
H355	28	3 B^7^ + 1 weak B^allo^	2 Type I + 1 B^allo^ + 1 Type II	1 B^allo^	B^7^/B^7^	7 strong	9.93 ± 0.01

† *From Jang et al. (2013)*;

‡ *where possible, in parentheses, the Type (for 5S^1^ rDNA) and genomic origin of all chromosomes carrying rDNA loci is identified from the amount and presence of PaB6. Superscripts indicate chromosome carrying locus; ^§^inferred from phylogenetic tree (Figures 3, 4)*;

¶ *analyzed by GISH. A, genome A; B^7^, genome B^7^; B^6^, genome B^6^; Type I, single locus of 5S^1^ rDNA on chromosome 1; Type II, duplicated locus of 5S^1^ rDNA on chromosome 1; B^allo^, rDNA loci found in Group I allotetraploids and derivatives*.

### Plant material

*Prospero autumnale* plants were collected in nature and cultivated in the Botanical Garden, University of Vienna (Table 1, Table S1). Every individual used was karyotyped, due to the high levels of chromosomal polymorphism. Chromosome numbers and karyotypes were assessed by standard Feulgen staining of meristematic root cells (Jang et al., 2013). Anthers in young flower buds fixed in ethanol: chloroform: acetic acid (6: 3: 1) and stored at −20°C were used for meiotic analyses.

Karyotypes were assembled in Corel Photo-Paint X5 (Figure S1) and idiograms based on at least three well-spread metaphase plates per individual were constructed. Idiograms of each polyploid cytotype based on 5S and 35S rDNA and *PaB6* satellite DNA FISH signals (see below) have been constructed with the program Autoidiogram (courtesy of Dr. Wolfgang Harand, formerly University of Vienna; see Weiss-Schneeweiss et al., 2008).

### Fluorescence *in situ* hybridization (FISH) and genomic *in situ* hybridization (GISH)

Chromosomes for FISH and GISH were prepared by enzymatic digestion and squashing as described in Jang et al. (2013). Briefly, meristems were digested with 1% cellulase Onozuka (Serva, Heidelberg, Germany), 1% cytohelicase (Sigma-Aldrich, Vienna, Austria), and 1% pectolyase (Sigma-Aldrich, Vienna, Austria), and squashed in 60% acetic acid. Cover slips were removed at −80°C and preparations air-dried.

Probes used for FISH were: a monomer of satellite DNA *PaB6* (Emadzade et al., 2014) isolated from the B^6^ genome of *P. autumnale* in plasmid pGEM-T easy, 35S rDNA (18S/25S rDNA) from *Arabidopsis thaliana* in plasmid pSK+, and the genic region of 5S rDNA from *Melampodium montanum* in plasmid pGEM-T easy. Probes were labeled with biotin- or digoxigenin-conjugated dUTPs (Roche, Vienna, Austria) by PCR (5S rDNA and satellite DNA *PaB6*) or using a nick translation kit (35S rDNA; Roche, Vienna, Austria). Digoxigenin was detected with antidigoxigenin conjugated with FITC (5 μg/ml; Roche, Vienna, Austria) and biotin with ExtrAvidin conjugated with Cy3 (2 μg/ml; Sigma-Aldrich, Vienna, Austria).

Total genomic DNAs from diploid cytotypes AA, B^6^B^6^, and B^7^B^7^ were isolated using the CTAB method (Doyle and Doyle, 1987; Jang and Weiss-Schneeweiss, 2015), sheared at 98°C for 5 min, and labeled with digoxigenin or biotin using a nick translation kit (Roche, Vienna, Austria). ff-GISH (formamide-free GISH) was carried out following Jang and Weiss-Schneeweiss (2015). The hybridization mix included 10% (w/v) dextran sulfate, 0.07×SSC, 1% (w/v) SS (salmon sperm DNA) and c. 125 μg/ml of each genomic probe (either biotin- or digoxigenin-labeled). After hybridization, slides were washed three times in 2×SSC at 42°C. Probes were detected using antidigoxigenin conjugated with FITC (digoxigenin), or ExtraAvidin conjugated with Cy3 (biotin). Chromosomal DNA was counterstained with DAPI (4′, 6-diamidino-2-phenylindole) and mounted in Vectashield antifade medium (Vector Laboratories, CA, USA).

Chromosomes were analyzed with an AxioImager M2 epifluorescent microscope, images acquired with a CCD camera, and files processed using AxioVision ver. 4.8 (Carl Zeiss, Vienna, Austria) with only those functions that apply to all images equally. A minimum of 20 well-spread metaphases and prometaphases were analyzed in each individual.

### DNA amplification, sequencing and the phylogenetic approach

Total genomic DNA was extracted from silica gel-dried leaf material as described in Jang et al. (2013). The internal transcribed spacer (ITS) region of nuclear 35S rDNA was amplified and sequenced using universal ITS primers (ITS 18sF and ITS 26sR) following the protocol of Jang et al. (2013). Three plastid regions were amplified using primers and protocols of Shaw et al. (2007; *ndh*A, *psb*D-*trn*T) and Demesure et al. (1995; *trn*D-*trn*T). All sequences are deposited in GenBank (accession numbers in Table S1).

PCR products were sequenced using dye terminator chemistry (Life Technologies, Vienna, Austria) and sequences were assembled and manually aligned as described in Jang et al. (2013). Three analyzed plastid regions were concatenated for the analyses. Indels were coded as binary characters following the “modified complex coding method” using SeqState version 1.36 (Müller, 2005), and the dataset with coded gaps was used in all analyses. A heuristic search for the most parsimonious (MP) trees was performed using PAUP 4.0.b10 (Swofford, 2002). The analyses involved 1,000 replicates of random sequence addition, with tree bisection–reconnection (TBR) branch swapping, saving no more than 10 trees per replicate. All characters were equally weighted and treated as unordered. Strict consensus trees were computed from all equally most parsimonious trees. Nodal support was assessed via bootstrapping (BS; Felsenstein, 1985) in PAUP^*^ 4.0b10 with 10,000 bootstrap replicates, each with 10 random sequence addition replicates holding maximally 10 trees per replicate, SPR branch swapping, and MulTrees on.

Maximum likelihood (ML) analyses were conducted for ITS and the concatenated three plastid regions using raxmlGUI 1.3 (Silvestro and Michalak, 2010) with the GTR+GAMMA nucleotide substitution model. The ML tree and BS for each region were obtained using the rapid bootstrap algorithm (Stamatakis et al., 2008) with 1,000 replicates.

### Genome size estimation

Genome size of 25 polyploid individuals (leaf material for two individuals was not available) of the *P. autumnale* complex was determined by flow cytometry with *Pisum sativum* “Kleine Rheinländerin” (1C = 4.42 pg, Greilhuber and Ebert, 1994) or *Solanum pseudocapsicum* (1C = 1.30 pg, Temsch et al., 2010) as the internal standard as described in Jang et al. (2013). Each individual (except for two) was measured three times. Measurements were done with a CyFlow ML flow cytometer (Partec, Muenster, Germany) equipped with a green laser (100 mW, 532 nm, Cobolt AB, Sweden) and 1C values were calculated according to the assumed linear fluorescence intensity relationship of both object and standard nuclei. CVs of all measurements were usually lower than 5% (Greilhuber et al., 2007) and never exceeded 10%.

## Results

### Genomic characterization of tetraploids

**Figure 1:**
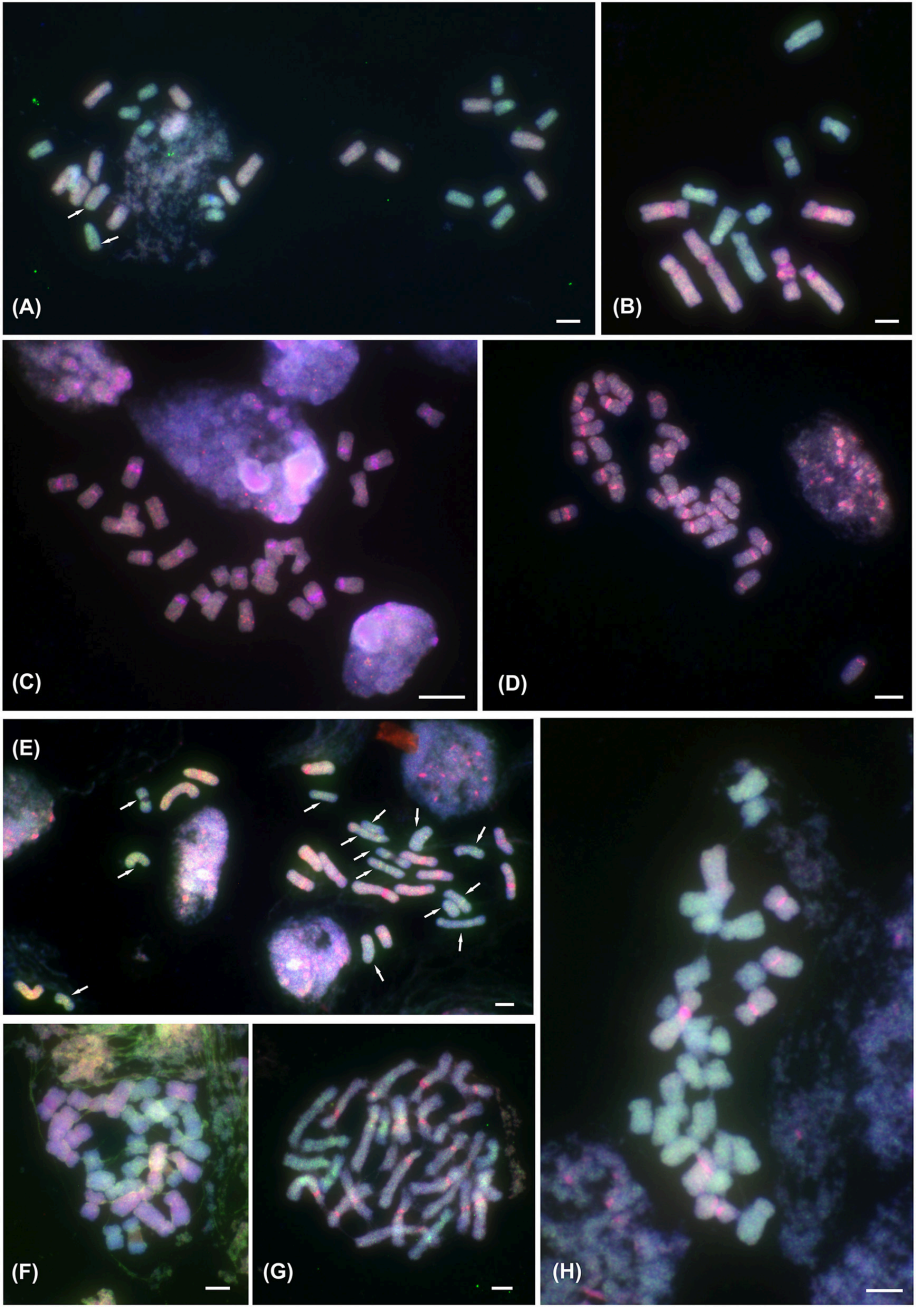
ff-GISH of the *Prospero autumnale* complex to mitotic metaphase chromosomes. **(A)** Localization of A (red; pale blue due to DAPI counterstaining) and B^7^ (green) genomic DNA (gDNA) in allotetraploid AAB^7^B^7^ (H603); 14 chromosomes labeled with gDNA of genome A and 14 with gDNA from genome B^7^; arrows indicate small intergenomic exchanges. **(B–H)** ff-GISH with B^6^ gDNA (red in **B–E**,**G,H**) or Group I allotetraploid gDNA (red in **F**) and B^7^ gDNA (green) in diploid and tetraploid hybrids of B^6^ and B^7^ origin: **(B)** Diploid homoploid hybrid B^6^B^7^ (2*n* = 13; H364); strong pericentric pink signals correspond to satDNA *PaB6* loci. **(C,D)** Group I allotetraploids: **(C)** 2*n* = 26 (H96), **(D)** 2*n* = 28 (H300). **(E)** Group II allotetraploid, 2*n* = 28 (H434); 14 chromosomes labeled with B^6^ gDNA (orange-red), and 14 with B^7^ gDNA (green; all arrowed); strong pericentric bands of satDNA *PaB6* in orange-red chromosomes. **(F)** Group II allotetraploid, 2*n* = 28 (H434) with 14 chromosomes labeled by Group I gDNA (red-pink with DAPI counterstain) and 14 with B^7^ gDNA (green). **(G,H)** Tertiary allotetraploids of B^6^ and B^7^ origin (Groups III and IV; 2*n* = 28; H238 and H152, respectively). **(G)** Group III, 2*n* = 28 (H238): 21 chromosomes labeled with B^6^ gDNA (red-pink) and 7 with B^7^ gDNA (green). **(H)** Group IV, 2*n* = 28 (H152): 7 chromosomes labeled with B^6^ gDNA (red-pink) and 21 with B^7^ gDNA (green). Plant number in brackets (see Table 1). Scale bar, 5 μm.

**Figure 2:**
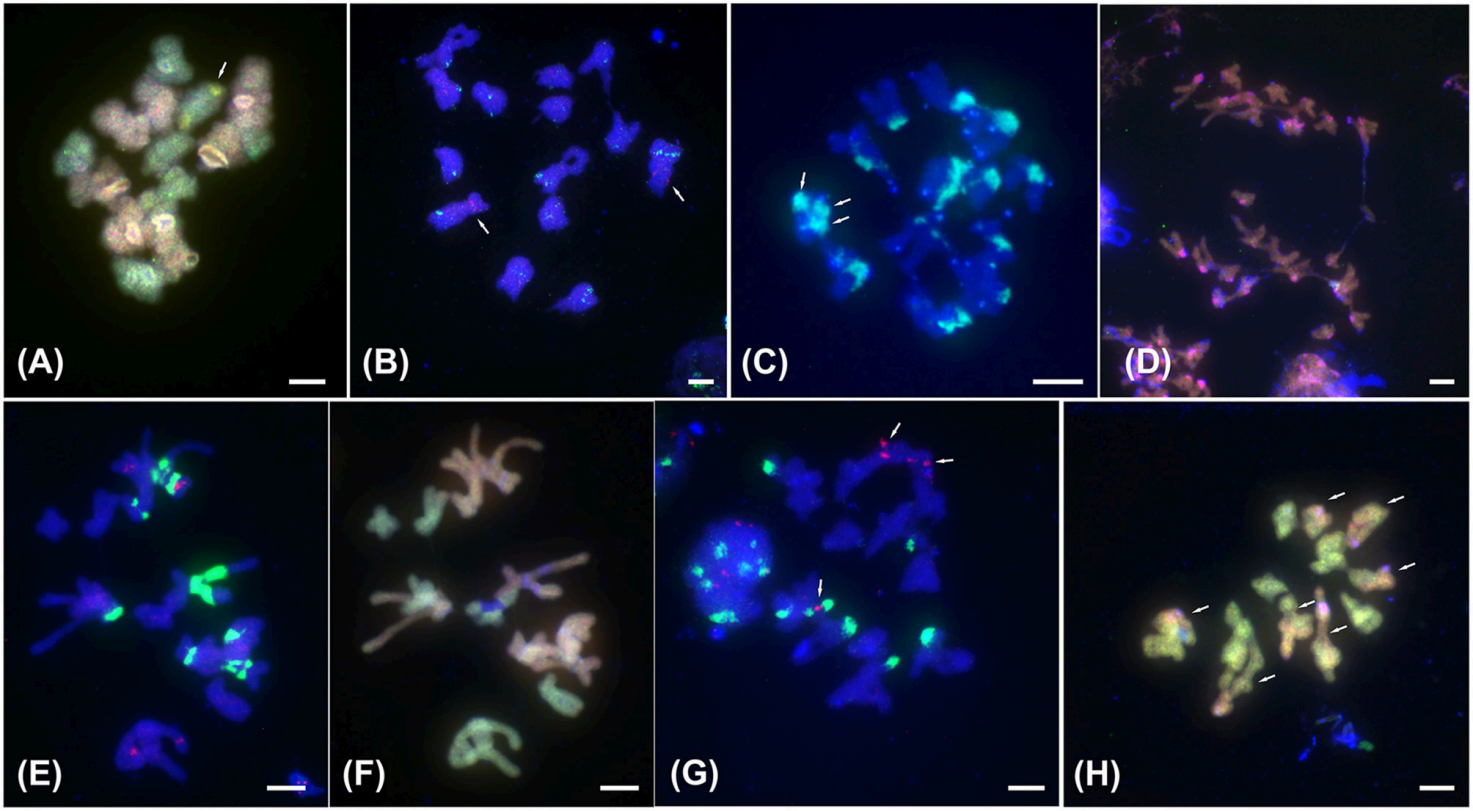
FISH with satellite DNA *PaB6* and 5S rDNA and ff-GISH with parental genomic DNAs to meiotic chromosomes of tetraploids of the *Prospero autumnale* complex. **(A)** ff-GISH of metaphase I bivalents of AAB^7^B^7^, 2*n* = 28 (H607): 7 A genome bivalents (red/pink) and 7 B^7^ bivalents (green); bivalent with 35S rDNA (yellow) arrowed. **(B)** B^7^B^7^B^7^B^7^ Type I, 2*n* = 28 (H534) with 14 bivalents hybridized with 5S rDNA (red, arrowed) and satellite DNA *PaB6* (green). **(C–H)** Allotetraploids of B^6^ and B^7^ origin. **(C)** Group I with 2*n* = 25 (H153): 12 bivalents and 1 trivalent. Arrows indicate three satellite DNA *PaB6* signals on trivalent (green-blue). **(D)** Group I with 2*n* = 25 (H153) at anaphase I: B^6^ (red) and B^7^ (green) genomic probes fail to distinguish parental genomes; strong pericentric signals reveal satDNA *PaB6* loci (pink). **(E,F)** Group II, 2*n* = 28 (H434): **(E)** labeled with *PaB6* (green) and 5S rDNA (pink) probes, **(F)** reprobed with B^6^ (orange) and B^7^ gDNAs (green) revealing seven Group I bivalents and seven B^7^ bivalents. Blue regions are unlabeled 35S rDNA sites. **(G,H)** Group IV with *2n* = 28 (H152): **(G)** 14 bivalents labeled with 5S rDNA (red, arrowed) and satellite DNA *PaB6* (green). **(H)** 7 homologous bivalents labeled with B^7^ gDNA (green) and 7 homoeologous bivalents labeled in part with B^7^ and in part with B^6^ gDNAs (orange; arrowed). Plant number in brackets (see Table 1). Scale bar, 5 μm.

Chromosome numbers and karyotypes, including localization of rDNAs and satDNA *PaB6* using FISH, were established for 27 tetraploids (Figures S1–S3), each one representing a different natural population (Table S1), with genome sizes available for 25 of them (Table 1). 11 individuals were analyzed by GISH (Table 1, Figures 1, 2). All results are summarized in **Figure 4**.

### Autotetraploids

Only genome B^7^ diploids were found to form autotetraploids (2*n* = 4*x* = 28). Both sublineages of the B^7^ diploids, type I and II, differing in the number of 5S rDNA loci formed respective polyploids. While distribution patterns of 5S and 35S rDNA, and of satDNA *PaB6* loci in the polyploids were additive compared to the diploid progenitors (Table 1, Figures S2A,B, S3A–C, Supplementary File S1), genome size of polyploids often deviated from the expected additive value, experiencing both upsizing (up to 12%; Type I B^7^ autotetraploids) and downsizing (−16%; Type I B^7^ autotetraploids; Table 1). Meiosis in these autotetraploids was regular and only bivalents were observed (Figure 2B).

### Allotetraploids

Three diploid cytotypes were involved in the formation of two genomic types of allotetraploids. Allotetraploids originating from diploid cytotypes A and B^7^ were chromosomally stable (2*n* = 28) showing additive number and distribution of 5S rDNA loci and of *PaB6* (Figures S1, S2C, S3D), a reduced number of 35S rDNA loci (loss from the AA subgenome), and slightly increased genome size compared to the expected value (5–8%; Table 1). In addition to visibly different sizes of chromosomes in the complement of the AAB^7^B^7^ allotetraploid reflecting different sizes of chromosomes in the parental diploid lineages, the parentage of these allotetraploids could be confirmed by ff-GISH (Figure 1A). Meiosis was regular with only bivalents observed at metaphase I, even in a plant with a small intergenomic translocation (Figure 2A).

In contrast to AAB^7^B^7^ allotetraploids, those originating from diploid cytotypes B^6^ and B^7^ had varying chromosome numbers from 2*n* = 25 to 2*n* = 28 (Table 1; Figure S1). This resulted from variation in the number of submetacentric fusion chromosome F^1^(6–7), of B^6^ origin, and free chromosomes 6 and 7 of B^7^ origin (Figure S7), and all plants were genetically balanced and had genome sizes equal to or somewhat larger than expected (up to 14%; Table 1). Whereas allotetraploids with 2*n* = 25, 26, or 27 could be identified by chromosome number and presence of one to three copies of fusion chromosome F^1^(6–7), those with 2*n* = 28, which may be confused with B^7^ autotetraploids, could be identified by the presence of 5S^2^ rDNA locus on the short arm of at least one chromosome B^6^2 (Table 1), coupled with strongly amplified *PaB6* signals on 7, 14, or 21 chromosomes (Figures S2D–J, S3E–K), features jointly occurring otherwise only in B^6^B^6^ diploids (Table 1). Within these allotetraploids, four distinct groups (I–IV) were identified, based on 5S rDNA, 35S rDNA and *PaB6* distributions, and GISH patterns. None of the individuals exhibited a strictly additive pattern of rDNA loci/signal number predicted from the diploid parents, but had instead experienced signal gain (prevalent for 5S rDNA) or loss (prevalent for the 35S rDNA locus; Table 1). Satellite DNA *PaB6* had either been amplified or showed additivity.

Group I allotetraploids had chromosome numbers of 2*n* = 25, 26, 27, or 28. They all possessed a strong pericentromeric *PaB6* signal on each chromosome (Figures S3E–H, S4A), although occasionally two or four were somewhat weaker. They had identical 5S and 35S rDNA signal numbers and distribution patterns, with an interstitial 35S rDNA locus on all chromosomes 3, Type I 5S^1^ rDNA signals on all chromosomes 1 and 5S rDNA (5S^2^) on all chromosomes 2 (Figures S2D–G; Supplementary File S1). Using ff-GISH with genomic DNA from B^6^ and B^7^ diploids failed to resolve the parental genomes, regardless of the chromosome number (2*n* = 26 and 28; Figures 1C,D, Figure S6). Meiosis, analyzed in individual with 2*n* = 25, was regular with bivalent formation, except for one trivalent of fused and free chromosomes 6 and 7 (Figures 2C,D, Figure S7). As ff-GISH analysis failed to differentiate parental genomes in Group I, the nature of meiotic bivalent pairing could not be established.

Plants belonging to Groups II–IV all had 2*n* = 28, lacked the fusion chromosome F^1^(6–7), and carried 7 (Group IV), 14 (Group II) or 21 chromosomes (Group III) marked by strongly amplified satDNA *PaB6* (Figures S1, S3I–K, S4B–F). The number of chromosomes 2 carrying a 5S^2^ rDNA locus correlated with the number of sets of chromosomes with strong *PaB6* signals in a ratio of one chromosome 2 with a 5S^2^ rDNA locus per seven *PaB6*-carrying chromosomes (Figures S3I–K). Additionally, one (Groups III and IV) or two (Group II) chromosomes 1 possessed a duplicated 5S^1^ rDNA locus as found in Type II B^7^ diploid and its autopolyploid derivative (see Supplementary File S1 for a more detailed description; Figures S2H–J, S3I–K). Using ff-GISH with gDNA of B^6^ and B^7^ diploids allowed parental chromosomes to be identified in all Groups II-IV tetraploids (all 2*n* = 28, Figures 1F–H) similarly to B^6^B^7^ hybrid (2*n* = 13; Figure 1B). In the same fashion both gDNA from B^7^ diploids of Type II and from Group I tetraploids (2*n* = 28) each labeled 14 chromosomes in Group II tetraploids (Figure 1H). Plants of Groups II and IV (Group III could not be analyzed) showed bivalent pairing, with strictly homologous pairing in Group II and mixed homologous and homoeologous pairing in Group IV (Figures 2E–H).

### Molecular phylogenetic analyses of cpDNA and nrITS sequences

Direct sequencing of ITS1+2 regions resulted in one ribotype in all but Group I (H331) and Group III (H238) individual of B^6^B^6^B^7^B^7^ allotetraploids. Cloning recovered both parental ribotypes in these two plants (Figure S5).

**Figure 3:**
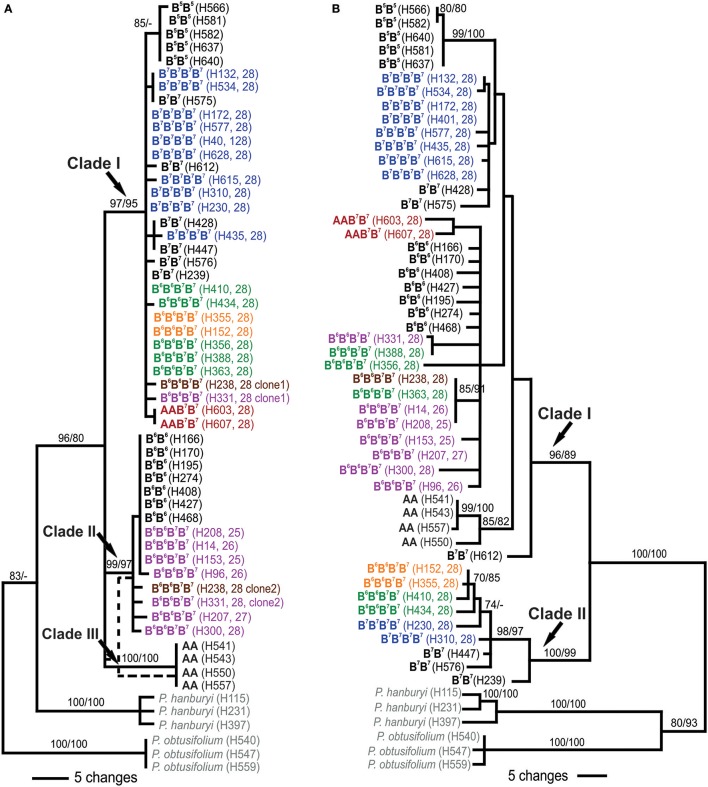
Phylogenetic relationships of diploids and polyploids of the *Prospero autumnale* complex inferred from ITS **(A)** and plastid **(B)** sequence data. Bootstrap support is given above branches as maximum parsimony/maximum likelihood. Information on genome composition (bold; except for outgroups in gray), plant number (in brackets) and chromosome number (2*n*; for polyploids) is provided for each analyzed individual (see Table 1 and Table S1 for details). Allopolyploids of AAB^7^B^7^ indicated in red, allopolyploids of B^6^ and B^7^ in purple (Group I), green (Group II), brown (Group III), and orange (Group IV), autotetraploids B^7^B^7^B^7^B^7^ in blue.

Phylogenetic analyses of ITS involved sequence data of 28 diploids and 27 polyploids, representing all *P. autumnale* cytotypes. Diploids were recovered in three clades (Figure 3A): Clade I of cytotype B^7^B^7^ and, nested therein, a monophyletic subclade of B^5^B^5^, Clade II involving cytotype B^6^B^6^, and Clade III with cytotype AA (bootstrap support [BS] from maximum parsimony [MP] and maximum likelihood [ML] for all clades 97–100/95–100). All B^7^B^7^B^7^B^7^ autotetraploids and AAB^7^B^7^ allotetraploids were found within Clade I (BS 97/95). B^6^B^6^B^7^B^7^ allotetraploids were recovered in two clades: Group I individuals in Clade II (BS 99/97), and individuals of Groups II and IV in Clade I (BS 97/95). The two individuals carrying both parental ribotypes (Groups I and III) were recovered in both Clades I and II.

Phylogenetic analyses of three plastid DNA markers grouped diploids into two clades (Figure 3B): Clade I (BS 96/89) containing three accessions of cytotype B^7^B^7^ and, as subclades, B^5^B^5^ (BS 99/100), B^6^B^6^ (BS <50/<50) and AA individuals (BS 85/82), and Clade II (BS 100/99) with the remaining three B^7^B^7^ plants. Amongst tetraploids, B^7^B^7^B^7^B^7^ grouped with B^7^B^7^ diploids in Clades I and II (Figure 3B), while AAB^7^B^7^ plants were recovered as a distinct subclade (BS 85/82) in Clade I, yet not close to AA diploids. B^6^B^6^B^7^B^7^plants occurred in Clades I and II. Group I and Group III, with three Group II plants, were recovered in a poorly supported subclade of Clade I that also contained B^6^B^6^ diploids, suggesting that this was the maternal parent. The remaining four plants—two each of Groups II and IV—grouped with B^7^B^7^ in Clade II.

Group I and IV plants have retained 35S rDNA on all four copies of the NOR-chromosome 3, and in these rDNA conversion was to the maternal ribotype. Group II has experienced complete, or near-complete, loss of two NORs from the Group I parent, regardless of whether it acted as maternal or paternal genome donor (Table 1, Figure 3).

## Discussion

**Figure 4:**
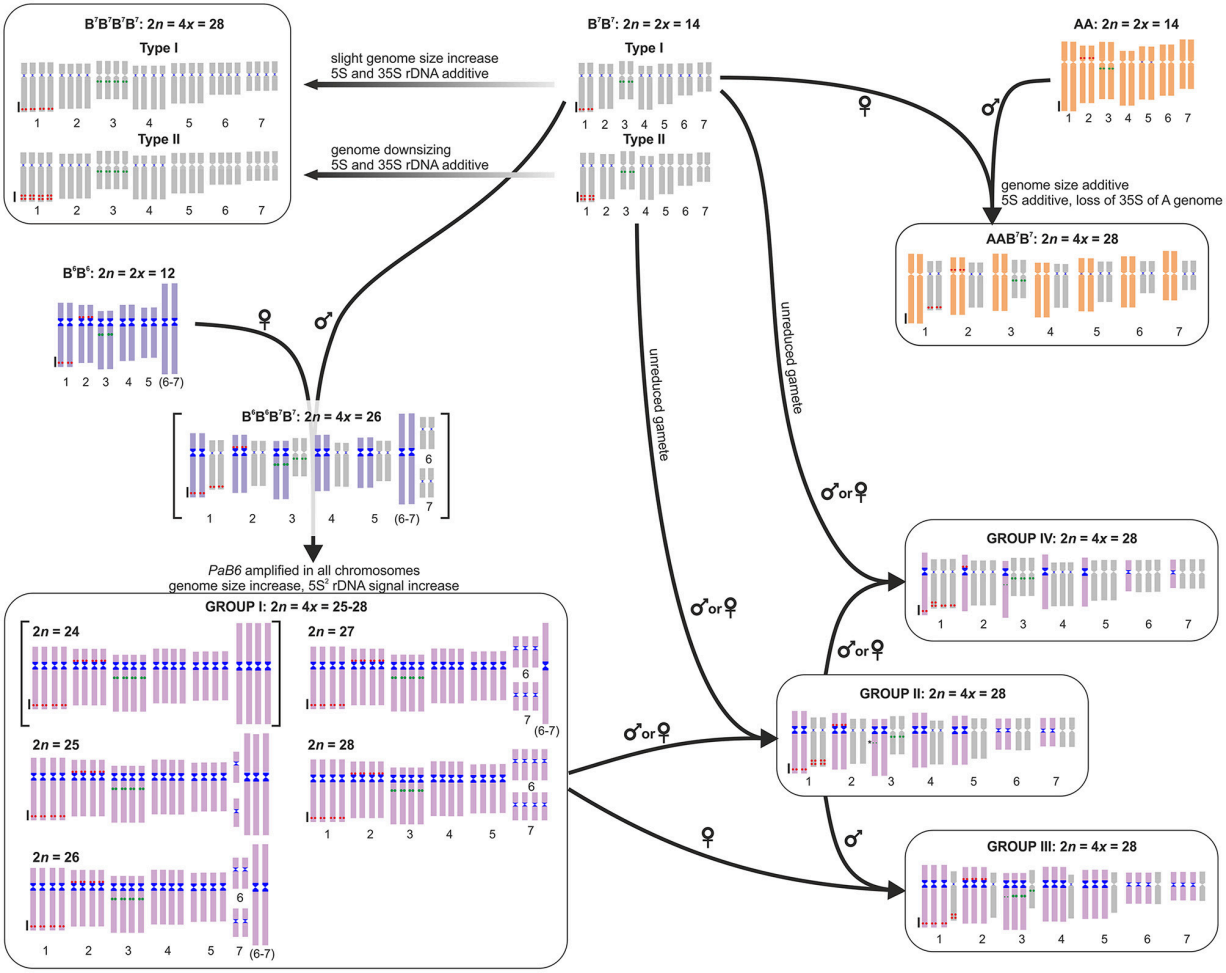
Model of the origin and evolution of polyploids in the *Prospero autumnale* complex. Diploid genomes represented by a standard idiogram, with 5S rDNA (red), 35S rDNA (green), and satellite DNA *PaB6* (blue). The B^6^B^6^B^7^B^7^ allotetraploid with 2*n* = 24 inferred but not found in nature is in square brackets. Parental subgenomes of Group I plants cannot be allocated to parents and are thus indicated by a single unique color. Group I chromosomes can be tracked in Groups II, III, and IV. Arrows indicate directions of crosses; inferred maternal and paternal parents are indicated.

*Prospero autumnale* contains a high diversity of tetraploids. Among those, autopolyploids (B^7^B^7^B^7^B^7^) formed at least twice (from Types I and II diploids: Figure 4; Ainsworth, 1980; Taylor, 1997; Jang et al., 2013), and likely more often given the large geographic area they occupy. These autopolyploids are following independent evolutionary trajectories with respect to genome size as well as copy and rDNA loci numbers and localization of satellite DNA *PaB6* although it is not yet clear whether this variation is geographically structured.

Allotetraploids of A/B^7^ and B^6^/B^7^ origin, despite sharing one parental genome, exhibit different evolutionary dynamics (Figure 4), possibly due to qualities of the second (different) parental genome. AAB^7^B^7^ tetraploids are meiotically stable, forming homologous bivalents at metaphase I, with multivalents, observed at zygotene resolved before chiasma formation (Jenkins et al., 1988; White et al., 1988). These polyploids have a genome size that is the sum of the parental genomes and lack gross genomic rearrangements, the only apparent change being a loss of 35S rDNA from the A paternal genome (Vaughan et al., 1993). Possibly the size difference between the parental chromosomes is sufficient to have prevented intergenomic meiotic interactions and genome homogenization in these tetraploids.

In contrast, allopolyploids involving B^6^ and B^7^ genomes, restricted to Crete where they intermix with their parents, are much more complex and form four groups (Figure 4). These groups result from genome homogenization, numerical convergence and nested cycles of hybridizations. Extant B^7^ and B^6^ diploid genomes differ not only in chromosome number but also in chromosome size and DNA amount. Newly formed tetraploids with 2*n* = 26 are expected to have a distinctly bimodal karyotype and a genome size being the sum of those of the parental genomes, but no plants like this have been found. Current Group I B^6^/B^7^ tetraploids deviate from a hypothetical inferred ancestral B^6^B^6^B^7^B^7^ tetraploid (Figure 4) in two ways. Firstly, there has likely been a spread of at least some B^6^-type repeat(s) throughout the B^7^ parental subgenome, as observed for satellite DNA *PaB6* characteristic of B^6^ genome (Emadzade et al., 2014), and subsequent genome homogenization, resulting in the failure to discriminate between parental genomes in ff-GISH analyses of Group I tetraploids and in non-additive patterns of genome size. The mechanism(s) and the extent of the involvement of *PaB6* and other repeat types in creating such homogenization remain unknown. Secondly, segregation from trivalents, formed of the F^1^(6–7) fusion chromosome (coming from B^6^) and the free chromosomes 6 and 7 (coming from B^7^) during meiosis, and random association of gametes generates numerically unbalanced (2*n* = 24–28), but genetically balanced offspring (Figure S7). Whereas chromosome numbers of 2*n* = 25, 26, 27, and 28 were found among only seven investigated individuals (Figure 4), no plant with 2*n* = 24 was found in this study nor in a previous large survey (Taylor, 1997), possibly indicating selection against such complements.

Group I tetraploids have been involved in nested rounds of hybridization (Figure 4). These probably involved the 2*n* = 28 race of Group I tetraploids, which is karyotypically indistinguishable from a B^7^ autotetraploid (numerical convergence). As Group II plants have two haploid chromosome sets nearly identical to Group I (although with near-suppression of Group I 35S rDNA) and two haploid sets identical to B^7^B^7^ Type II, they likely arose from a cross between those two and as Type II B^7^ autotetraploids are not known from Crete, such a cross would likely invoke unreduced gametes of Type II B^7^B^7^ diploids (Figure 4). Group II plants have regular meiosis, forming 14 meiotic homologous bivalents and they may correspond to tetraploids described as B^7^B^7^CC by Vaughan et al. (1997).

Groups III and IV have karyotypes comprising three sets of one genome and one set of another (derivatives of B^7^ and B^6^). Specifically, the Group III plant is the result of a cross between Group I and Group II (Figure 4), a hypothesis supported by data on rDNAs, *PaB6*, ff-GISH, and genome size. Similarly, Group IV originated from a cross between Group II and a B^7^B^7^ Type I diploid (likely via unreduced gametes, as autotetraploids of B^7^ cytotype are not known from Crete; Figure 4), a hypothesis supported by all molecular data and by meiotic configurations (seven homologous and seven homoeologous bivalents).

In *Prospero*, both genomic autopolyploids and allopolyploids form recurrently from the same or genomically very similar diploid parents. These tetraploids with distinctive origins can cross and backcross at different ploidy levels to produce an even wider spectrum of genotypes (Figure 4). This might provide high levels of genetic variation upon which selection can act and within which drift may occur. However, extensive and rampant reticulation may prevent or at least impede population isolation, diversification and, finally, radiation and fixation of polyploid cytotypes, thus contributing to a “lag phase” between polyploid formation and species diversification and/or speciation, akin to the lag-time model for angiosperm lineages (Schranz et al., 2012; Tank et al., 2015; Dodsworth et al., 2016). Therefore, only once new polyploid cytotypes are fixed and sufficiently isolated, polyploidy will confer allelic diversity and fixed heterozygosity allowing generation of new metabolic and biochemical networks and neo- and sub-functionalization of genes. These genetic processes may generate further isolation barriers between populations (Le Comber et al., 2010) and allow taxa to occupy and exploit new niches (Soltis et al., 2016), thus enhancing the frequency of diversification and radiation of polyploids.

## Conclusions

The intricacies that have been described here in the cytologically diverse *Prospero autumnale* complex involving diploid divergence combined with polyploidisation and rounds of hybridization have resulted in distinct genomically differentiated tetraploids. Remarkably, this astonishing genomic diversification has remained cryptic, as it has occurred without conspicuous morphological differentiation (Jang, 2013). This study demonstrates the power of molecular cytogenetic approaches in disentangling the complex history of post-polyploidisation genomic diversification. All tetraploids of the *Prospero autumnale* complex are part of a dynamic hybrid swarm that contains a perfect cocktail of features that promote permanent establishment and further diversification of polyploids: multiple, recurrent and widespread origins that might accommodate for local extinctions, local diversifications and diverse allele inheritance, as well as high levels of genetic variation upon which selection can act. Thus, unlike some neopolyploid systems, where genetic instability often confers risk of extinction or genetic bottlenecks (e.g., Abbott and Forbes, 2002) *Prospero autumnale* might possess all ingredients for establishment of new polyploids.

## Author contributions

HW-S, T-SJ, and JP: conceived and coordinated the study; T-SJ, KE, and ET: performed research; T-SJ, HW-S, KE, and ET: analyzed the data; HW-S, T-SJ, JP, and AL: interpreted the data and wrote the paper with input from KE and ET. All authors read and approved the final manuscript.

### Conflict of interest statement

The authors declare that the research was conducted in the absence of any commercial or financial relationships that could be construed as a potential conflict of interest.
