# Supplementary material for: Multiple Origins and Nested Cycles of Hybridization Result in High Tetraploid Diversity in the Monocot Prospero
Source: Front Plant Sci. 2018 Apr 6;9:433. doi: 10.3389/fpls.2018.00433 (PMC5932365; doi:10.3389/fpls.2018.00433)

Supplementary Figure S1. Karyotypes of selected auto- and allotetraploids in the *Prospero autumnale* complex analysed in this study. Plant number in brackets (see Table 1). Scale bar, 5µm.

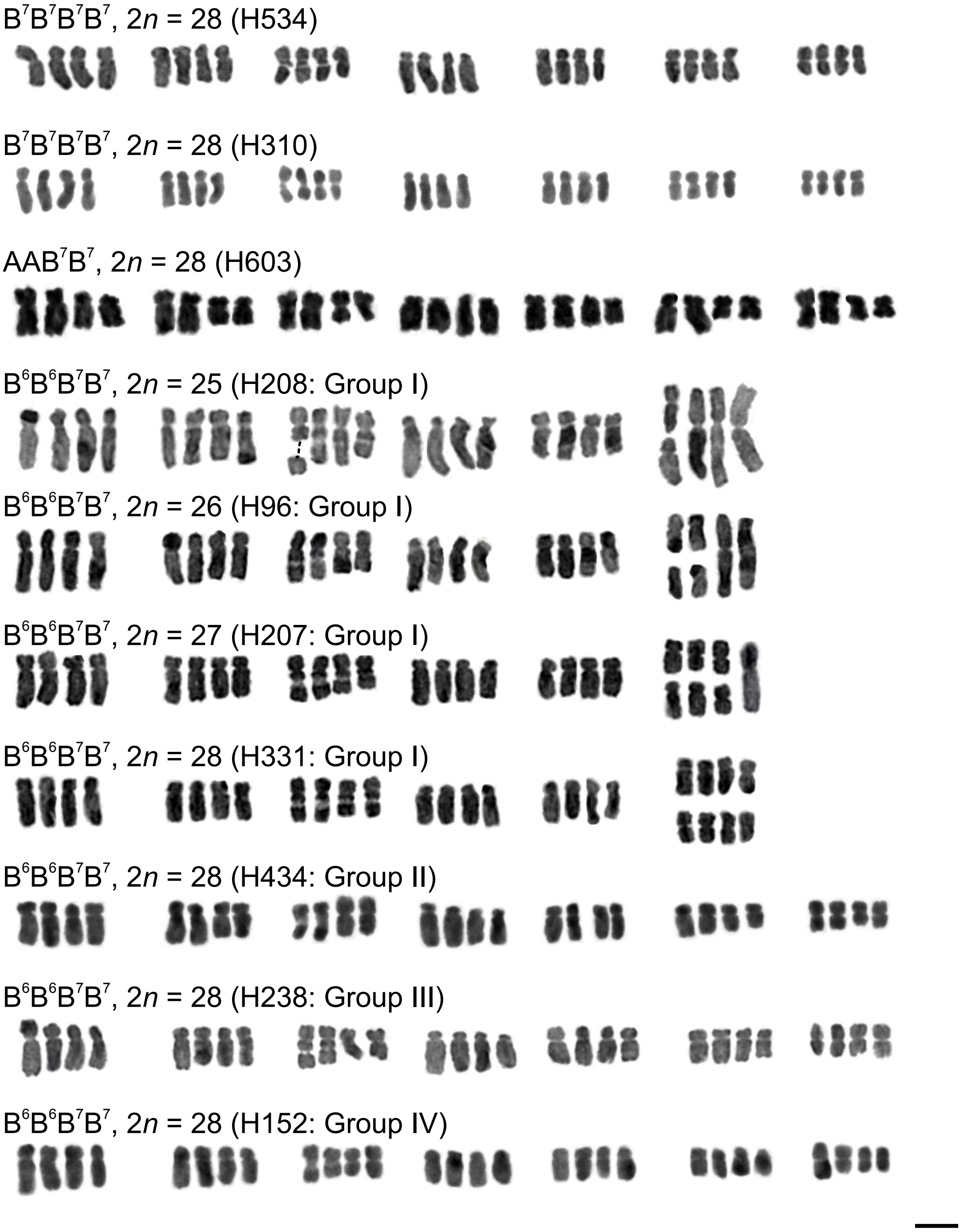

Supplement: Supplementary file 1 [file Image1.PDF]
