# Supplementary material for: Multiple Origins and Nested Cycles of Hybridization Result in High Tetraploid Diversity in the Monocot Prospero
Source: Front Plant Sci. 2018 Apr 6;9:433. doi: 10.3389/fpls.2018.00433 (PMC5932365; doi:10.3389/fpls.2018.00433)
Supplement: Supplementary file 2 [file Image2.PDF]

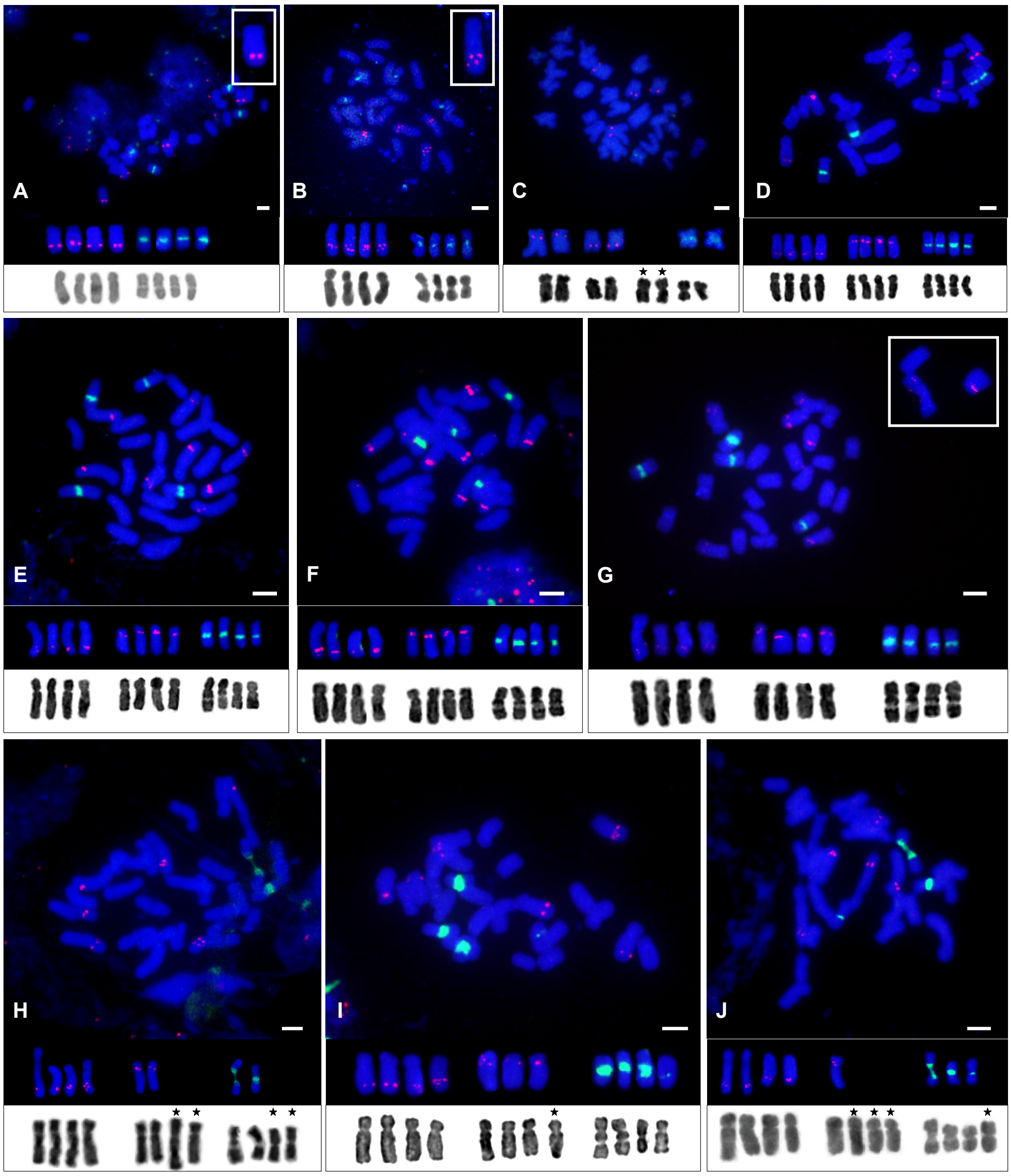

Supplementary Figure S2. Localization of 5S (red) and 35S rDNA (green) using FISH on mitotic chromosomes of tetraploids of the *Prospero autumnale* complex. rDNA loci abbreviations in Table 1. **(A, B)** $B^7B^7B^7B^7$, 2*n* = 28: **(A)** Type I $5S^1$ rDNA locus (H577), **(B)** Type II $5S^1$rDNA locus (H310); see also Fig. 1b. **(c)** AAB^7B^7 (H603). **(D-J)**: allotetraploids of B^6 and B^7 origin: **(D-G)**, Group I: **(D)** 2*n* = 25 (H153), **(E)** 2*n* = 26 (H14), **(F)** 2*n* = 27 (H207), **(G)** 2*n* = 28 (H331); **(H)** Group II, 2*n* = 28 (H363). **(I)** Group III, 2*n* = 28 (H238); **(J)** Group IV, 2*n* = 28 (H152). Asterisks against chromosomes stained with the Feulgen method indicate those lacking 5S or 35S rDNA signals (see Supplementary Figure S1). **(A, B)** Inserts show a Type I and a Type II $5S^1$ rDNA locus; **(G)** Insert: chromosomes lying outside main group in high magnification. Plant number in brackets (see Table 1). Scale bar, 5 µm.
