# Supplementary material for: Multiple Origins and Nested Cycles of Hybridization Result in High Tetraploid Diversity in the Monocot Prospero
Source: Front Plant Sci. 2018 Apr 6;9:433. doi: 10.3389/fpls.2018.00433 (PMC5932365; doi:10.3389/fpls.2018.00433)
Supplement: Supplementary file 3 [file Image3.PDF]

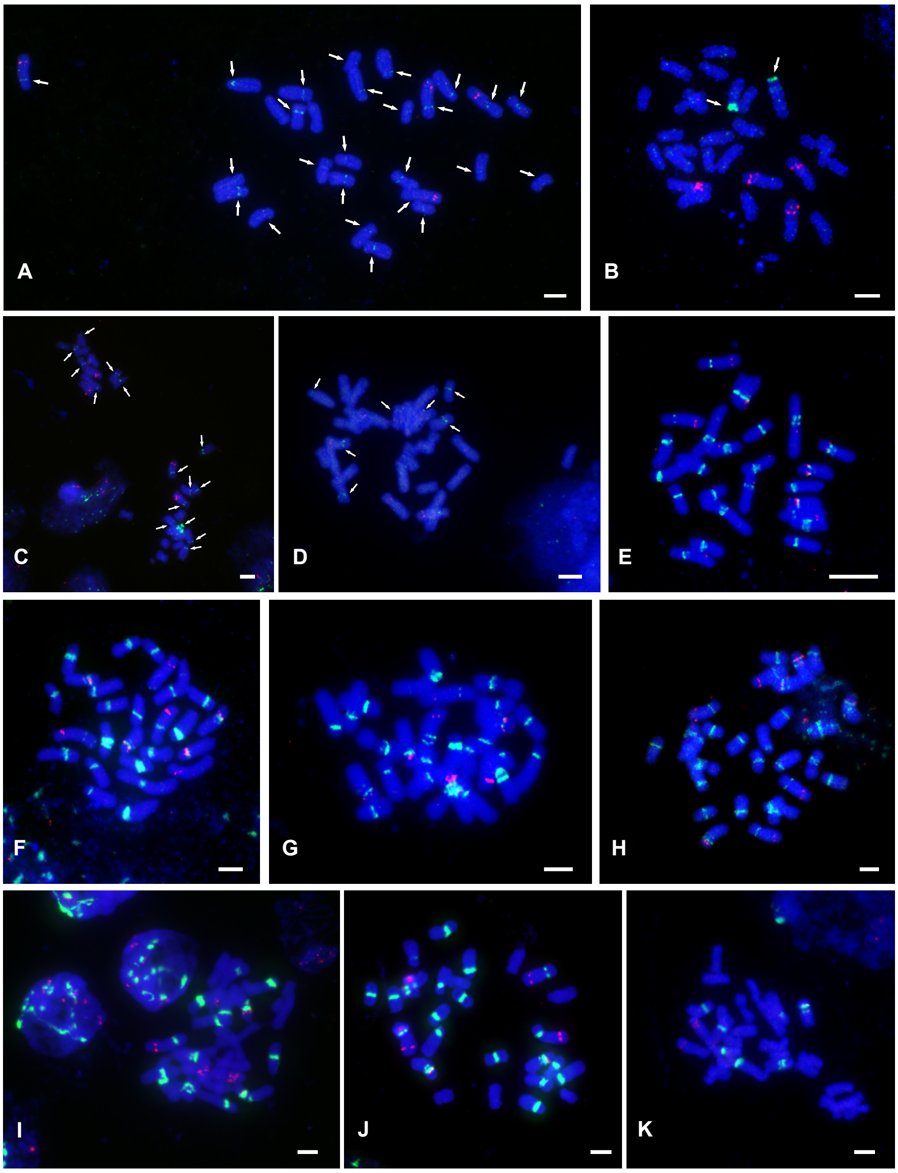


**Supplementary Figure S3. Localization of 5S rDNA (red) and satellite DNA *PaB6* (green) in tetraploids of the *Prospero autumnale* complex. (A-C)** $B^7B^7B^7B^7$,2*n* = 28: **(A)** Type I $5S^1$ rDNA locus (H534). **(B)** Type II $5S^1$ rDNA locus (H310). **(C)** Type II $5S^1$ rDNA locus (H230). **(D)** $AAB^7B^7$ (H603). **(E-K)** $B^6B^6B^7B^7$: **(E-H)** Group I, *PaB6* amplified in all chromosomes: **(E)** 2*n* = 25 (H153). **(F)** 2*n* = 26 (H14). **(G)** 2*n* = 27 (H207). **(H)** 2*n* = 28 (H331). **(I)** Group II, 14 strong *PaB6* signals (H363). **(J)** Group III, 21 strong *PaB6* signals (H238). **(K)** Group IV, 7 strong *PaB6* signals (H152). Arrows indicate weak satellite DNA *PaB6* signals. Plant number in brackets (see Table 1). Scale bar, 5 μm.
