# Supplementary material for: Multiple Origins and Nested Cycles of Hybridization Result in High Tetraploid Diversity in the Monocot Prospero
Source: Front Plant Sci. 2018 Apr 6;9:433. doi: 10.3389/fpls.2018.00433 (PMC5932365; doi:10.3389/fpls.2018.00433)

Supplementary Figure S4. FISH with satellite DNA *PaB6* in B^6/B^7 allotetraploids in the *Prospero autumnale* complex allows parental origin of chromosomes carrying 5S and 35S rDNA to be determined: **(A)** Group I (H208), 2*n* = 25: 35S rDNA (green) and *PaB6* satellite (red) with 22 strong and 3 weak signals ; **(B-F)** Plants with 2*n* = 28. **(B)** Group II (H434), 35S rDNA (green) in two chromosomes 3 lacking *PaB6* signals (red), contributed by maternal B^7 genome. **(C)** Group II (H363), two strong 35S rDNA signals (green) in chromosomes 3 from B^7 paternal genome (lacking *PaB6*) with one weak 35S rDNA (arrow in inset) in one of two maternal chromosomes 3 exhibiting strong *PaB6* signals. **(D, E)** Group III (H238), the same cell after reprobing: **(D)** 5S rDNA (green) and 35S rDNA (red; arrow indicating weak signal), **(E)** 5S rDNA (green) and *PaB6* (red); parental origin of all rDNA bearing chromosomes can be inferred (see also Table 1). **(F)** Group IV (H152), three 35S rDNA signals (green) in three chromosomes 3 contributed by maternal genome B^7, with one weak 35S rDNA signal (arrow) contributed by paternal parent (*PaB6* in red, Table 1). Plant number in brackets (see Table 1). Scale bar, 5 µm.

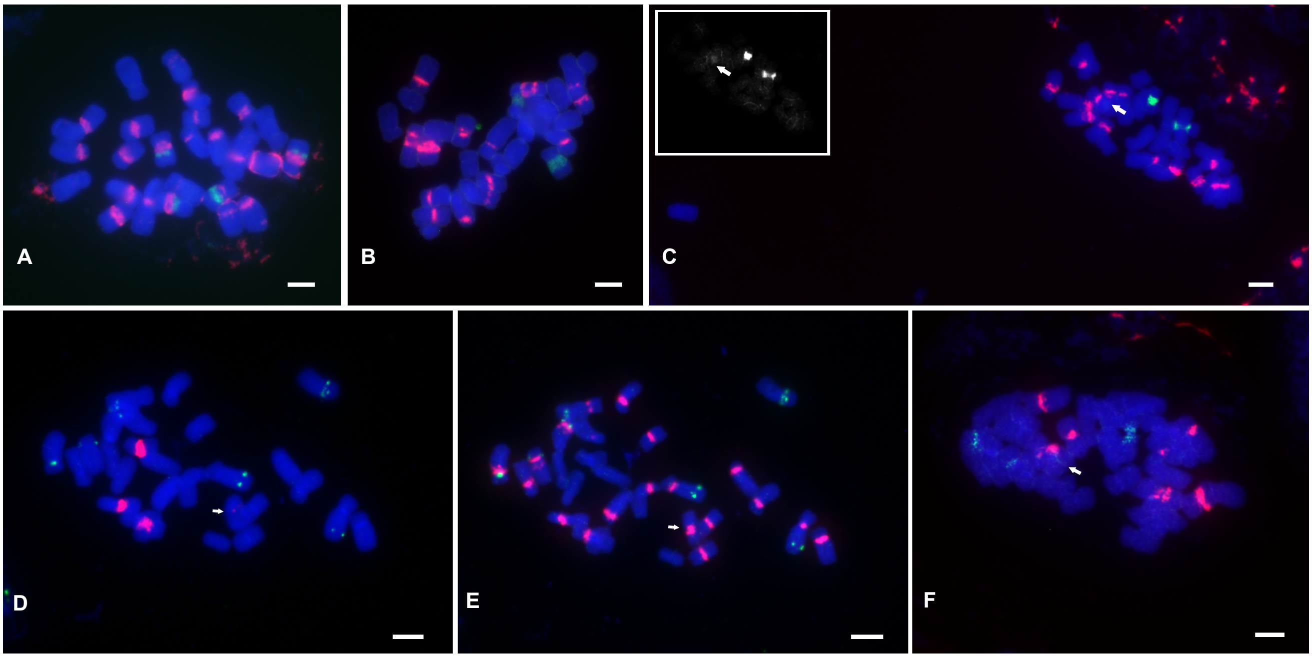

Supplement: Supplementary file 4 [file Image4.PDF]
