# Supplementary material for: Multiple Origins and Nested Cycles of Hybridization Result in High Tetraploid Diversity in the Monocot Prospero
Source: Front Plant Sci. 2018 Apr 6;9:433. doi: 10.3389/fpls.2018.00433 (PMC5932365; doi:10.3389/fpls.2018.00433)
Supplement: Supplementary file 5 [file Image5.PDF]

Supplementary Figure S5. Variable (parsimony informative) nucleotide positions in ITS (35S rDNA) sequence alignment.

Diploids AA, B^{7}B^7 and allotetraploids AAB^{7}B^7

position in alignment

	108	194	295	320	331	333	458	551	559	560	575	621	641	657	665	731
P. autumnale H541 AA	C	T	T	T	T	A	C	T	T	C	C	A	A	C	T	C
P. autumnale H543 AA	C	T	T	T	T	A	C	T	T	C	C	A	A	C	T	C
P. autumnale H550 AA	C	T	T	T	T	A	C	T	T	C	C	A	A	C	T	C
P. autumnale H557 AA	C	T	T	T	T	A	C	T	T	C	C	A	A	C	T	C
P. autumnale H603 AAB7B7	T	C	C	C	G	G	T	-	-	-	T	C	G	A	A	G
P. autumnale H607 AAB7B7	T	C	C	C	G	G	T	-	-	-	T	C	G	A	A	G
P. autumnale H447 B7B7	T	C	A	C	G	G	T	-	-	-	T	C	G	A	A	G
P. autumnale H575 B7B7	T	C	C	C	G	G	T	-	-	-	T	C	G	A	A	G
P. autumnale H576 B7B7	T	C	C	C	G	G	T	-	-	-	T	C	G	A	A	G
P. autumnale H239 B7B7	T	C	C	C	G	G	T	-	-	-	T	C	G	A	A	G
P. autumnale H428 B7B7	T	C	A	C	G	G	T	-	-	-	T	C	G	A	A	G
P. autumnale_H612_B7B7	T	C	C	C	G	G	T	-	-	-	T	C	G	A	A	G

Diploids B^{7}B^7 and autotetraploids B^{7}B^{7}B^{7}B^7

	395	411
P. autumnale H132 B7B7B7B7	C	T
P. autumnale H534 B7B7B7B7	C	T
P. autumnale H172 B7B7B7B7	C	G
P. autumnale H230 B7B7B7B7	C	G
P. autumnale H310 B7B7B7B7	C	G
P. autumnale H401 B7B7B7B7	C	G
P. autumnale H435 B7B7B7B7	A	G
P. autumnale H577 B7B7B7B7	C	G
P. autumnale H615 B7B7B7B7	C	G
P. autumnale H628 B7B7B7B7	C	G
P. autumnale H447 B7B7	A	G
P. autumnale H575 B7B7	C	T
P. autumnale H576 B7B7	C	G
P. autumnale H239 B7B7	C	G
P. autumnale H428 B7B7	A	G
P. autumnale_H612_B7B7	C	G

Diploids B^{7}B^7, B^{6}B^6 and allotetraploids of B^7 and B^6 origin

	108	187	228	316	331	360	575	610	632	641	665	736
P. autumnale H239 B7B7	T	G	T	C	G	C	T	G	C	G	A	G
P. autumnale H428 B7B7	T	G	T	C	G	C	T	G	C	G	A	G
P. autumnale H447 B7B7	T	G	T	C	G	C	T	G	C	G	A	G
P. autumnale H575 B7B7	T	G	T	C	G	C	T	G	C	G	A	G
P. autumnale H576 B7B7	T	G	T	C	G	C	T	G	C	G	A	G
P. autumnale H612 B7B7	T	G	T	C	G	C	T	G	C	G	A	G
P. autumnale H152 B6B6B7B7	T	G	T	C	G	C	T	G	C	G	A	G
P. autumnale H355 B6B6B7B7	T	G	T	C	G	C	T	G	C	G	A	G
P. autumnale H356 B6B6B7B7	T	G	T	C	G	C	T	G	C	G	A	G
P. autumnale H388 B6B6B7B7	T	G	T	C	G	C	T	G	C	G	A	G
P. autumnale H410 B6B6B7B7	T	G	T	C	G	C	T	G	C	G	A	G
P. autumnale H434 B6B6B7B7	T	G	T	C	G	C	T	G	C	G	A	G
P. autumnale H238 B6B6B7B7 clone1	T	G	T	C	G	C	T	G	C	G	A	G
P. autumnale H166 B6B6	C	T	C	C	T	A	C	A	T	A	T	C
P. autumnale H170 B6B6	C	T	C	C	T	A	C	A	T	A	T	C
P. autumnale H195 B6B6	C	T	C	C	T	A	C	A	T	A	T	C
P. autumnale H274 B6B6	C	T	C	C	T	A	C	A	T	A	T	C
P. autumnale H408 B6B6	C	T	C	C	T	A	C	A	T	A	T	C
P. autumnale H427 B6B6	C	T	C	C	T	A	C	A	T	A	T	C
P. autumnale H468 B6B6	C	T	C	C	T	A	C	A	T	A	T	C
P. autumnale H14 B6B6B7B7	C	T	C	C	T	A	C	A	T	A	T	C
P. autumnale H96 B6B6B7B7	C	T	C	C	T	A	C	A	T	A	T	C
P. autumnale H153 B6B6B7B7	C	T	C	C	T	A	C	A	T	A	T	C
P. autumnale H178 B6B6B7B7	C	T	C	C	T	A	C	A	T	A	T	C
P. autumnale H207 B6B6B7B7	C	T	C	T	T	C	C	A	T	A	T	C
P. autumnale H208 B6B6B7B7	C	T	C	C	T	A	C	A	T	A	T	C
P. autumnale H238 B6B6B7B7 clone2	C	T	C	T	T	C	C	A	T	A	T	C
P. autumnale H300 B6B6B7B7	C	T	C	T	T	C	C	A	T	A	T	C
P. autumnale H363 B6B6B7B7	C	T	C	C	T	A	C	A	T	A	T	C
P. autumnale H238 B6B6B7B7	Y	K	Y	Y	K	C	Y	R	Y	R	W	S
P. autumnale_H331_B6B6B7B7	Y	K	Y	Y	K	C	Y	R	Y	R	W	S
