# Supplementary material for: Multiple Origins and Nested Cycles of Hybridization Result in High Tetraploid Diversity in the Monocot Prospero
Source: Front Plant Sci. 2018 Apr 6;9:433. doi: 10.3389/fpls.2018.00433 (PMC5932365; doi:10.3389/fpls.2018.00433)

Supplementary Figure S6: GISH images (from Figure 1) as individual channels and a merged image. Scale bar, 5 µm.

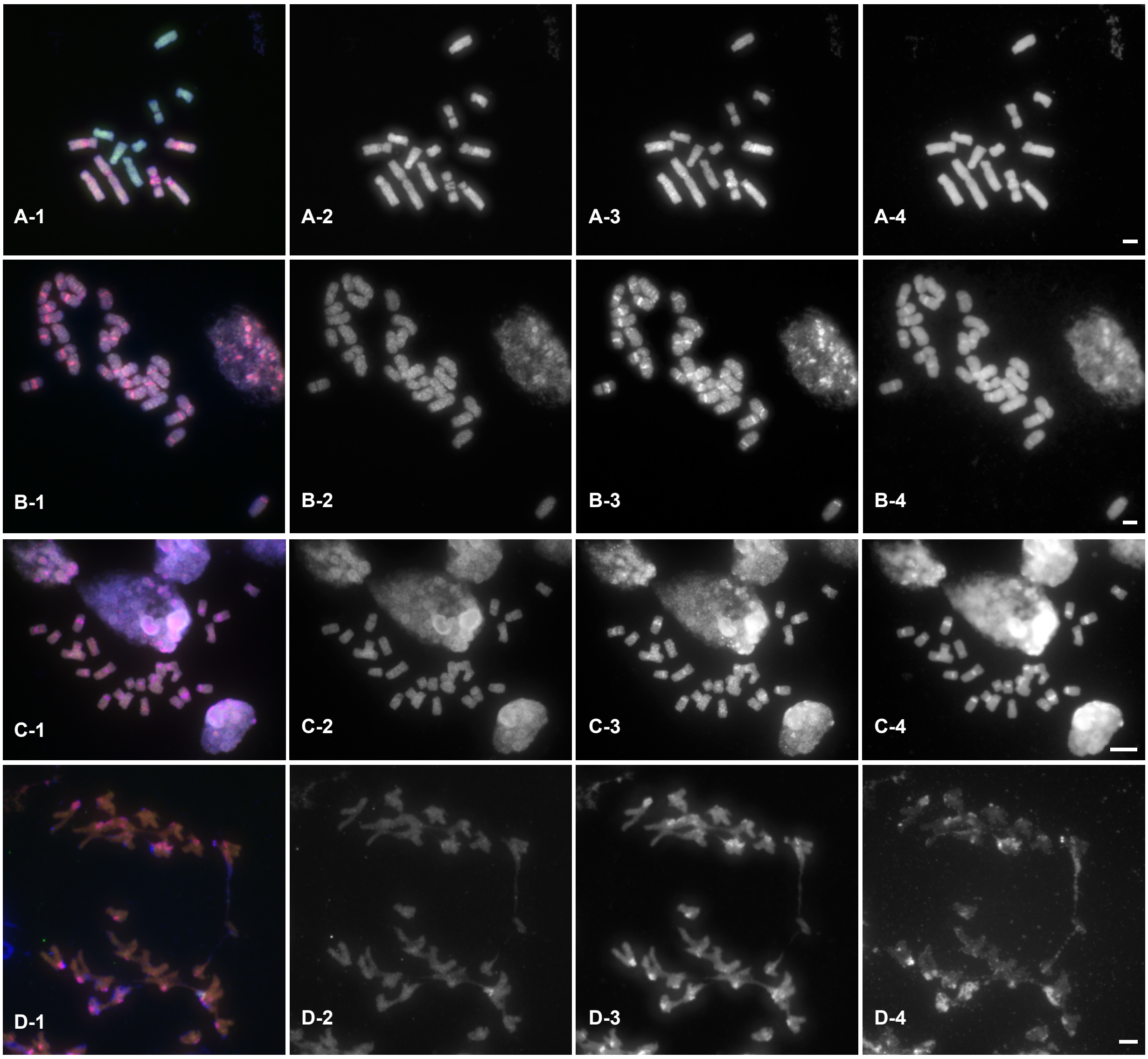

Supplement: Supplementary file 6 [file Image6.PDF]
