# Supplementary material for: Multiple Origins and Nested Cycles of Hybridization Result in High Tetraploid Diversity in the Monocot Prospero
Source: Front Plant Sci. 2018 Apr 6;9:433. doi: 10.3389/fpls.2018.00433 (PMC5932365; doi:10.3389/fpls.2018.00433)
Supplement: Supplementary file 7 [file Image7.PDF]

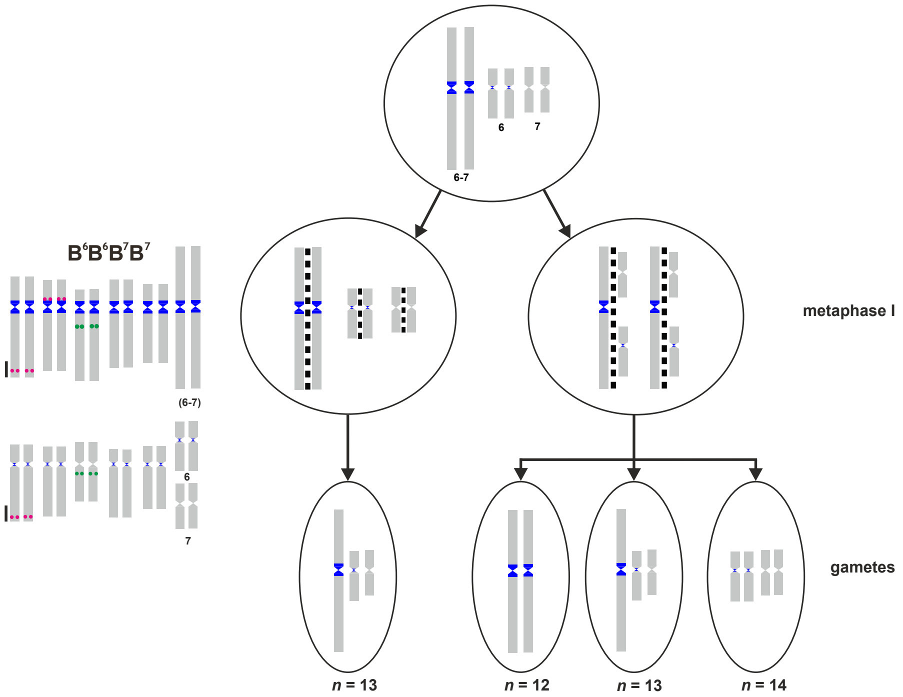

Supplementary Figure S7. Meiotic pairing behaviour resulting in numerical variation in $B^6B^6B^7B^7$ allotetraploids of Group I. Left: *in silico* karyotype of $B^6B^6B^7B^7$ allotetraploid, $2n = 26$. Right: only pairing and segregation patterns of chromosomes 6, 7 and fusion chromosome F^1(6-7) are indicated, since these lead to numerical variation in gametes. From top to bottom: set of chromosomes 6, 7 and fusion chromosome F^1(6-7); pairing in meiosis I; combination of these chromosomes in gametes.
