# Supplementary material for: Multiple Origins and Nested Cycles of Hybridization Result in High Tetraploid Diversity in the Monocot Prospero
Source: Front Plant Sci. 2018 Apr 6;9:433. doi: 10.3389/fpls.2018.00433 (PMC5932365; doi:10.3389/fpls.2018.00433)
Supplement: Supplementary file 8 [file Table1.PDF]

Table S1. Plant material of *Prospero* studied with collection details, chromosomes numbers, and GenBank accession numbers (ITS and plastid DNA). ITS data for *P. obtusifolium*, *P. hanburyi* and diploids of *P. autumnale* complex from Jang et al. (2013).

Cytotype	Locality; Collection; accession number	2*n*	GenBank accession numbers
			ITS / *ndh*A, *trn*T-*psb*D, *trn*D-*trn*Y
Prospero obtusifolium	Spain; Parker; H540	8	KC899275 / KF941354, KF897714, KF897656
	Spain; Parker; H559	8	KC899272 / KF941356, KF897716, KF897658
	Morocco; Parker; H547	8	KC899273 / KF941355, KF897715, KF897657
P. hanburyi	Turkey, Findikpinar; Speta; H115	14	KC899269 / KF941351, KF897711, KF897653
	Turkey, Findikpinar; Speta; H397	14	KC899271 / KF941353, KF897713, KF897655
	Turkey, Narlikuyu; Silifke; H231	14	KC899270 / KF941352, KF897712, KF897654
P. *autumnale* complex			
Diploids			
AA	Spain, Huelva; Parker; H541	14	KC899278 / KF941398, KF897758, KF897700
	Spain, Huelva; Parker; H557	14	KC899282 / KF941402, KF897761, KF897704
	Spain, Badajoz; Parker; H543	14	KC899279 / KF941399, KF897759, KF897701
	Portugal, Peniche; Parker; H550	14	KC899281 / KF941401, KF897760, KF897703
B^7B^7 (Type I $5S^1$ rDNA)	Greece, Naxos; Speta; H575	14	KC899300 / KF941375, KF897738, KF897677
	Israel, Nene Han; Parker; H612	14	KC899301 / KF941378, KF897742, KF897680
	Italy, Sicily; Speta; H428	14	KC899298 / KF941368, KF897731, KF897670
	No locality; Speta; H447	14	KC899299 / KF941371, KF897734, KF897673
	Cyprus; Speta; H239	14	KC899297 / KF941365, KF897728, KF897667
B^7B^7 (Type II $5S^1$ rDNA)	Serbia, Siget-Baun; Rat; H576	14	KC899303 / KF941376, KF897739, KF897678
B^6B^6	Greece, Crete; Speta; H166	12	KC899284 / KF941384, KF897723, KF897686
	Greece, Crete; Speta; H170	12	KC899285 / KF941385, KF897724, KF897687
	Greece, Crete; Speta; H195	12	KC899290 / KF941386, KF897726, KF897688

	Greece, Crete; Speta; H274	12	KC899291 / KF941387, KF897747, KF897689
	Greece, Crete; Jahn; H408	12	KC899288 / KF941388, KF897748, KF897690
	Greece, Crete; Jahn & Böhling; H427	12	KC899293 / KF941389, KF897749, KF897691
	Greece, Crete; Speta; H468	12	KC899292 / KF941390, KF897750, KF897692
B^5B^5	Libya, Mt. Tobi; Parker; H566	10	KC899313 / KF941392, KF897752, KF897694
	Libya, Mt. Tobi; Parker; H581	10	KC899314 / KF941393, KF897753, KF897695
	Libya, Mt. Tobi; Parker; H582	10	KC899316 / KF941394, KF897754, KF897696
	Libya, Mt. Tobi; Parker; H637	10	KC899312 / KF941396, KF897756, KF897698
	Libya, Nagasa; Parker; H640	10	KC899315 / KF941397, KF897757, KF897699
Polyploids			
AAB^7B^7	Portugal, Algarve; Parker; H603	28	KF873571 / KF941431, KF941485, KF941458
	Portugal, Algarve; Parker; H607	28	KF873572 / KF941432, KF941486, KF941459
$B^6B^6B^7B^7$	Greece, Crete; Speta; H153	25	KF873550 / KF941412, KF941466, KF941439
	Greece, Crete; Weigl; H208	25	KF873553 / KF941415, KF941469, KF941442
	Greece, Crete; Speta; H14	26	KF873548 / KF941410, KF941464, KF941437
	Greece, Crete; Speta; H96	26	KF873575 / KF941435, KF941489, KF941462
	Greece, Crete; Speta; H207	27	KF873552 / KF941414, KF941468, KF941441
	Greece, Crete; Speta; H152	28	KF873549 / KF941411, KF941465, KF941438
	Greece, Crete; Speta; H238	28	KF873556, KF873555 / KF941417, KF941471, KF941444
	Greece, Crete; Speta; H300	28	KF873557 / KF941418, KF941472, KF941445
	Greece, Crete; Raus; H331	28	KF873560, KF873559 / KF941420, KF941474, KF941447
	Greece, Crete; Speta; H355	28	KF873561 / KF941421, KF941475, KF941448
	Greece, Crete; Passauer; H356	28	KF873562 / KF941422, KF941476, KF941449
	Greece, Crete; Böhling; H363	28	KF873563 / KF941423, KF941477, KF941450
	Greece, Crete; Raus; H388	28	KF873564 / KF941424, KF941478, KF941451

	Greece, Crete; Jahn; H410	28	KF873566 / KF941426, KF941480, KF941453
	Greece, Crete; Speta; H434	28	KF873567 / KF941427, KF941481, KF941454
$B^7B^7B^7B^7$	Croatia, Kornati; Parker; H628	28	KF873574 / KF941434, KF941488, KF941461
	France, Morbihan; Ragot; H615	28	KF873573 / KF941433, KF941487, KF941460
	Greece, Euboea; Speta; H230	28	KF873554 / KF941416, KF941470, KF941443
	Greece, Kos; Speta; H310	28	KF873558 / KF941419, KF941473, KF941446
	Greece, Zakinthos; Speta; H132	28	KF873547 / KF941409, KF941463, KF941436
	Italy, Sicily; Speta; H435	28	KF873568 / KF941428, KF941482, KF941455
	Italy, Sardinia; Grims; H534	28	KF873569 / KF941429, KF941483, KF941456
	Montenegro, Kotor; Rat; H577	28	KF873570 / KF941430, KF941484, KF941457
	Republic of Malta; Speta; H401	28	KF873565 / KF941425, KF941479, KF941452
	Spain, Minorca; Speta; H172	28	KF873551 / KF941413, KF941467, KF941440
