# Supplementary material for: Multiple Origins and Nested Cycles of Hybridization Result in High Tetraploid Diversity in the Monocot Prospero
Source: Front Plant Sci. 2018 Apr 6;9:433. doi: 10.3389/fpls.2018.00433 (PMC5932365; doi:10.3389/fpls.2018.00433)
Supplement: Supplementary file 9 [file DataSheet1.DOCX]

**Supplementary File S1.** Results: distribution of satDNA *PaB6*, 5S rDNA and 35S rDNA loci (Supplementary Figs 2 and 3).

**Autotetraploids**

All had four 35S rDNA sites, located on the long arm near the centromere of chromosome 3 (Table 1; Figure S2a–b), and a 5S^1^ rDNA locus on all four chromosomes 1. Eight plants were homozygous for the Type I 5S^1^ locus (single locus), while two were homozygous for the Type II 5S^1^ locus (duplication, Figures S2a–b, S3a–c). As in B^7^B^7^ diploids, satellite DNA *PaB6* was present in low copy numbers, with weak or very weak signals in pericentric regions of a variable number of chromosomes, thus a range is given (Table 1; Figure S3a–c). In Type I autotetraploids, *PaB6* signals were seen on 15 to 26 chromosomes (Figure S3a), the expected additive pattern from diploids (Table 1). The two Type II tetraploid plants showed very few *PaB6* signals, as did their progenitor diploids, but had one or two novel strong distal *PaB6* signals (Figure S3b–c), located either on chromosome 2 or the morphologically similar chromosome 5 (Figure S1).

**Autotetraploids**

AAB^7^B^7^ allotetraploids showed only two 35S rDNA signals, in chromosomes of B^7^ origin (Table 1; Figure S2c). 5S rDNA site number and localization, by contrast, was additive, with an interstitial signal on the short arm of chromosomes A2 (5S^2^), and a distal signal on the long arm of chromosomes B^7^1 (5S^1^; Figure S2c). The distribution and amount of *PaB6* corresponded to diploid additive values (Figure S3d).

DNA contents of allotetraploids AAB^7^B^7^ (2*n* = 28) were 12.69 and 13.1 pg (Table 1), slightly larger than the additive parental value (5-8%; Table 1). Parental genome sizes were reflected in visibly different sizes of chromosomes in the complement. The A3 chromosomes in tetraploids lacked NORs and 35S rDNA (Figure S1).

**B6B7**

**Group I** allotetraploids had chromosome numbers of 2*n* = 25, 26, 27 or 28. They all possessed a strong pericentromeric *PaB6* signal on each chromosome (Figures S3e–h, S4a), although occasionally two or four were somewhat weaker. They had identical 5S and 35S rDNA signal numbers and distribution patterns: (i) an interstitial 35S rDNA locus on all four chromosomes 3; (ii) Type I 5S^1^ rDNA signals on long arms of all chromosomes 1; (iii) 5S rDNA (5S^2^) signals on short arms of all four chromosomes 2, although only two were expected.

**Group II** tetraploids had (i) strong *PaB6* signals on 14 of the 28 chromosomes (7 pairs; Figures S2h, S3i); (ii) three chromosome pairs with 5S rDNA - one pair typical of chromosome B^6^1, one pair typical of chromosome B^7^1 Type II with no detectable *PaB6* signals, and the third pair on chromosome B^6^2 with strong *PaB6* signals; (iii) strong 35S rDNA signals on two chromosomes 3 without *PaB6* signals (Figure S4b). Three plants also carried a weak 35S rDNA signal on one of the two chromosomes 3 with a strong *PaB6* signal (Figures S3i, S4c).

The single **Group III** tetraploid had strong *PaB6* signals on 21 chromosomes, comprising three sets of chromosomes 1–7 (Figures S2i, S3j). 5S rDNA loci were found on all four copies of chromosomes 1: one carried a B^7^ Type II signal and a weak *PaB6* signal, while the remaining three carried Type I 5S^1^ rDNA and strong *PaB6* signals (Figure S3j). Three chromosomes 2 had strong *PaB6* signals and 5S^2^ rDNA, while the fourth lacked 5S^2^ rDNA and had a very weak *PaB6* signal. All four chromosomes 3 carried 35S rDNA: two with a strong and one with a weak 35S rDNA but with strong *PaB6* signals, while the fourth chromosome had a strong 35S signal with a weak *PaB6* signal (Figure S4d-e).

The two **Group IV** plants had only seven chromosomes with strong *PaB6* signals, representing a single complement (Figures S2j, S3k). All four chromosomes 1 carried 5S^1^ rDNA signals but comprised three types - one with a strong *PaB6* signal and a Type I 5S^1^ rDNA locus, two lacking *PaB6* but with Type I 5S^1^ rDNA, and the fourth lacking *PaB6* but with a Type II 5S^1^ rDNA signal. One copy of chromosome 2 carried a 5S^2^ rDNA signal and strong *PaB6* signal while the other three lacked both. Strong 35S rDNA signals were detected in three chromosomes 3 lacking *PaB6*, while the fourth had a very weak 35S rDNA signal but strong *PaB6* (Figure S4f).
